# Fluid mixing optimization with reinforcement learning

**DOI:** 10.1038/s41598-022-18037-7

**Published:** 2022-08-22

**Authors:** Mikito Konishi, Masanobu Inubushi, Susumu Goto

**Affiliations:** 1 Graduate School of Engineering Science, Osaka University, Osaka, 560-8531 Japan; 2 Department of Applied Mathematics, Tokyo University of Science, Tokyo, 162-8601 Japan

**Keywords:** Fluid dynamics, Mechanical engineering, Engineering, Physics, Mathematics and computing, Applied mathematics, Computational science, Computer science

## Abstract

Fluid mixing is crucial in various industrial processes. In this study, focusing on the characteristics that reinforcement learning (RL) is suitable for global-in-time optimization, we propose utilizing RL for fluid mixing optimization of passive scalar fields. For the two-dimensional fluid mixing problem described by the advection–diffusion equations, a *trained* mixer realizes an exponentially fast mixing without any prior knowledge. The stretching and folding by the trained mixer around stagnation points are essential in the optimal mixing process. Furthermore, this study introduces a physically reasonable transfer learning method of the trained mixer: reusing a mixer trained at a certain Péclet number to the mixing problem at another Péclet number. Based on the optimization results of the laminar mixing, we discuss applications of the proposed method to industrial mixing problems, including turbulent mixing.

## Introduction

Fluid mixing plays a fundamental role in various industrial processes. However, most mixing processes are empirically designed by using trial-and-error methods through physical experiments, rather than mathematical optimization. Although turbulence is an “effective mixer”^1^, in some cases (e.g., a bioreactor or a mixer in food industry processes), turbulent mixing is not appropriate because strong shear flows damage the materials to be mixed. Moreover, sustaining turbulent flows in micro-mixers is difficult due to low Reynolds numbers; this necessitates enhanced mixing by laminar flows. Therefore, mixing optimization by laminar flows is crucial. Several analytical studies have evaluated the efficiency of laminar mixing protocols^2–5^, e.g., proving the exponential bounds of the mixing speed; however, the research on constructive optimization methods remains limited.

This study proposes a mixing optimization based on *reinforcement learning* (RL) as a constructive method. To illustrate the effectiveness of the RL algorithm for fluid mixing optimization, we first summarize its mathematical framework. The RL algorithm is formulated in terms of the Markov decision process (MDP)^6,7^: $M= \{ {S}, {A}, p_{0}, P, R\}$, where *S* denotes the set of states, ${S}=\{s_1, \cdots s_{|{S}|} \}$; *A* denotes the set of actions, ${A}=\{ a_{1}, \cdots a_{|{A}|} \}$; $p_{0}$ denotes the probability distribution of the initial state, $p_{0}: {S} \rightarrow [0,1]$; *P* denotes the transition probability, $P: {S} \times {S}\times {A} \rightarrow [0,1]$; and *R* denotes the reward function, $R:{S} \times {A} \rightarrow \mathbb {R}$. The initial state, $s_{0}$, is determined by $p_{0}(\cdot )$, and in the next step, the state is determined by the transition probability, $P(\cdot |s_{0},a_{0})$, which requires the action, $a_0$. The action is determined by the *policy*, $\pi : {S} \rightarrow {A}$, as $a=\pi (s)$. The RL algorithm is implemented to determine the optimal policy, $\pi ^*$, for the given MDP, which maximizes the expectation of the cumulative reward, $\sum _{t=0}^{\infty } \gamma ^{t} R_{t+1}$. Here, $\gamma \in (0,1)$ denotes the discount factor and $R_{t+1}:=R(s_{t},a_{t})$.

The RL algorithm maximizes the cumulative reward (i.e., global-in-time) rather than the instantaneous reward, $R_{t}$ (i.e., local-in-time). Therefore, it is suitable for global-in-time optimization problems. Designing efficient mixing protocols is one of the global-in-time optimization problems, as the final scalar field depends on the temporal order of actions in the entire mixing process, which includes stretching and folding by fluid flows and its coupling with molecular diffusion. An illustrative example was presented in *History matters* of Villermaux^8^. Despite the effectiveness of the RL algorithms in solving a diverse range of problems in fluid mechanics^9–11^, including nuclear fusion^12^ and turbulence modelling^13^, the fluid mixing problem remains unexplored.

The RL algorithm is suitable for the global-in-time optimization problems, but not for problems with a high-dimensional state space in general, which is known as the curse of dimensionality^6^. Particularly, the high dimensionality of the state space for fluid mixing renders the implementation of the RL algorithm challenging. This study investigates an optimization problem formulated by Mathew et al.^2^, in which the velocity field is given by the superposition of the prescribed fields. This reduces the dimension of the state space for the fluid motion to one^2^; a single parameter, denoted by $\theta$ later, determines the state of the fluid motion. This optimization problem was based on a physical experiment using the electromagnetically driven flow^14^. The conjugate gradient descent method was introduced as a prototype of the fluid mixing optimization^2^. To ensure that the RL algorithm can handle the flow field with a reduced degree of freedom, we focus on the same optimization problem.

In this paper, we demonstrate for the first time that the RL algorithm is suitable for fluid mixing optimizations. This algorithm identifies an effective flow control, which results in exponentially fast mixing without prior knowledge. The mechanisms behind efficient mixing are uncovered by focusing on the flow around the fixed points from the viewpoint of dynamical system theory^15,16^. This study also proposes an effective transfer learning method for the trained mixer by considering the diffusion effect on mixing. Based on the optimization results of the laminar mixing, we discuss applications of the proposed method to industrial mixing problems, including turbulent mixing, in the “Conclusion and discussion” section.

## Methods

### Fluid mixing optimization

**Figure 1:**
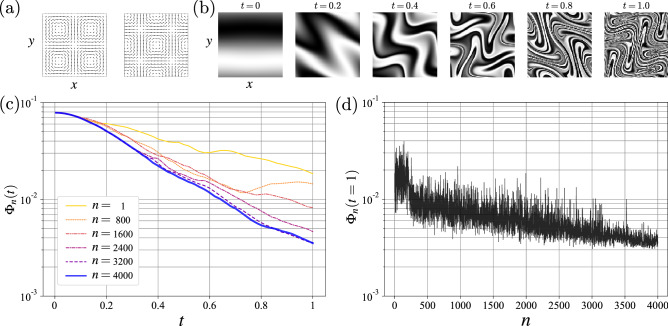
Optimization of fluid mixing using the reinforcement learning (RL) algorithm: (**a**) velocity vector fields of $u_{1}(x,y)$ (left) and $u_{2}(x,y)$ (right) on the two-dimensional torus, $\mathbb {T}^2$; (**b**) snapshots of the time evolution of scalar field, *c*(*x*, *t*), advected by the optimized flow; (**c**) mix-variance, $\Phi _{n}(t)~(t \in [0,1])$, for the *n*th episode ($n=1$, 800, 1600, 2400, 3200, and 4000); (**d**) mix-variance, $\Phi _{n}(t=1)$, at the end of each episode.

We consider the following optimization problem formulated by Mathew et al.^2^ as the benchmark problem, in which the velocity field, $u(x,y,t) = \alpha _{1}(t) u_{1}(x,y) + \alpha _{2}(t) u_{2}(x,y)$, is used. Here, $u_1(x,y) = (-\sin (2 \pi x)\cos (2 \pi y), \cos (2 \pi x) \sin (2 \pi y))$ and $u_2(x,y) = u_1(x-0.25,y-0.25)$ (see Fig. 1a). The time evolution of the passive scalar, *c*(*x*, *y*, *t*), is described by the advection–diffusion equations on the two-dimensional torus, $\mathbb {T}^2$ (the periodic boundary condition):

1 $$\begin{aligned} \partial _{t} c + u \cdot \nabla c = \frac{1}{\text {Pe}}\Delta c,~~~c(x,y,0)=c_{0}(x,y),~~~(x,y,t) \in \mathbb {T}^2 \times [0,1], \end{aligned}$$

where $\text {Pe} \in (0,\infty ]$ represents the Péclet number. As a constraint on the flow control, the time integral of the kinetic energy, $\frac{1}{2} \int _0^{1} \int _{\mathbb {T}^2} u^{2} d\mathbf{x} dt = \int _0^{1} \alpha _i(t) R_{ij} \alpha _j (t) dt =:\mathscr {E}$, is fixed, where $R_{ij}:= \frac{1}{2} \int _{\mathbb {T}^2} u_i \cdot u_j d\mathbf{x}~~(i=1,2,~j=1,2)$. We set $\alpha (t)=2\sqrt{\mathscr {E}} (\cos \theta (t), \sin \theta (t))$, by which the constraint is always satisfied. We also set $\mathscr {E}=1.25$ as in Mathew et al.^2^. In this problem, the velocity field, *u*(*x*, *y*, *t*), is determined by a single parameter, $\theta (t)$, referred to as the *flow parameter*.

The variance of the scalar field is often used to measure the degree of mixedness. However, as it is a conserved quantity in the absence of diffusion (i.e., $\frac{d}{dt} \int _{\mathbb {T}^2} c^{p}dx \equiv 0~~(\forall p \in \mathbb {N})$), it is unsuitable as a measure of the mixing process. Instead, we employ the mix-variance defined by $\Phi (c)=\Vert c \Vert ^2_{H^{-1/2}} := \sum _{k} \frac{1}{\sqrt{1+ (2 \pi \Vert k \Vert )^2}} | c_k |^2$, where $c_k$ denotes the Fourier coefficient of the scalar field^17^. The mix-variance is equivalent to the Mix-Norm that was originally introduced to characterize the multiscale property of the mixed scalar field^17^. Furthermore, Mathew et al.^17^ showed the equivalence between the decay of $\Phi (c)$, the weak convergence in $L^{2}$, and the mixing of ergodic dynamical systems in Theorem 3.2 (see also Lin et al.^3^ for the extension of the theorem). To summarize the optimization problem, we use the RL algorithm to determine the function, $\theta : [0,1] \rightarrow \mathbb {R}$, that minimizes the mix-variance at the end of the mixing process, $\Phi ( c (\cdot , t=1))$.

We conduct a numerical simulation of the advection–diffusion equations (Eq. 1) using the fourth-order Runge–Kutta scheme for the temporal integration with $\Delta t=0.001$ and the Fourier spectral method for spatial discretization with a grid of $250 \times 250$, which is the same as the one used in Mathew et al.^2^.

### Optimization of mixing with RL

Here, we consider the optimization of the action-value function (Q function) $Q^{\pi }(s,a):= \mathbb {E}[ \sum _{t=0}^{\infty } \gamma ^{t} R_{t+1}|S_{0}=s, A_{0}=a]$ instead of the policy $\pi$, and obtain the optimal Q function, $Q^*: {S} \times {A} \rightarrow \mathbb {R}$. The Banach fixed-point theorem mathematically ensures that such an optimal Q function exists as a fixed point of the Bellman operator^6,7^. We obtain the optimal policy as $\pi ^{*}(s) := \mathrm{argmax}_{a \in {A}} Q^{*}(s,a)$.

As a standard implementation of the RL algorithm, we employ the deep Q network^18^, which approximates the Q function by using the deep neural network denoted by $Q^w: \mathbb {R}^{N_s} \times {A} \rightarrow \mathbb {R}$. Here, $N_s$ and *w* denote the dimension of the state space and the connection weights in the neural network, respectively. The inputs to the network are the scalar field, *c*(*x*, *y*, *t*), and the velocity field, *u*(*x*, *y*, *t*). The values of these fields on $\mathbb {T}^2$ are observed on the square grid $83\times 83$, and the state, *s*, of the MDP is defined as the observed values of the velocity field, $u(x_{i}, y_{i}, t)~~(i =1,\ldots , N_O)$, where $N_O=83\times 83$, and those of the scalar fields over the last five steps; that is, $s_{t} := (u (x_{i},y_{i}, t), \{ c(x_{i}, y_{i}, \tau ) \}_{\tau = t,~t- \Delta t_{O},\ldots ,~t - 4 \Delta t_{O}}) \in \mathbb {R}^{N_s}$, and $\Delta t_O$ denotes the time interval of the successive observations. Therefore, the dimension of the state space is $N_s=7 \times N_O$. The network consists of four hidden layers, and each activation function is ReLU as Mnih et al.^18^. The discount factor is $\gamma =0.99$. The more details of the deep Q network structure and its implementations are described in the “Supplementary Information”. The initial distribution, $p_{0}$, is given by the delta function such that $\theta (0)=0$ and $c(x,y,0)=\sin (2 \pi y)$.

The time interval of the successive observations is $\Delta t_O=0.004$, which is the same value used in the benchmark problem^2^, and $\Delta t_Q=5 \Delta t_O$, where $\Delta t_Q$ denotes the time interval of the successive updates of the Q function. Hence, for each period of $\Delta t_Q$, the RL algorithm observes the scalar fields determined by the advection–diffusion equations (Eq. 1) with the fixed velocity field. Then, the Q function, i.e., the weights in the neural network, is updated. A single unit of *episode* corresponds to a single mixing process, i.e., solving the initial value problem of the advection–diffusion equations (Eq. 1) for $0 \le t \le 1$. The total number, $N_{e}$, of episodes for the training is $N_{e}=4000$. The results with the larger number of episodes, $N_{e} = 5000$, are qualitatively identical to those with $N_{e} = 4000$.

As the action, *A*, of the MDP, the RL algorithm can change the value of the flow parameter, $\theta (t)~~(0\le t\le 1)$. The velocity field, *u*(*x*, *y*, *t*), is determined by the single-parameter $\theta (t)$, and the flow control is realized by changing $\theta (t)$. The discretization of the temporal change of the flow parameter is $\theta (t + \Delta t_{Q}) = \theta (t) + \omega \Delta t_{Q}$ with $\omega \in \{ 0, \omega _{+}, \omega _{-}\}(={A})$, where $\omega _{+}=\pi /(4\Delta t_{Q})$ and $\omega _{-}=-\pi /(4\Delta t_{Q})$. The action, $\omega$, is selected following the $\varepsilon$-greedy method^6,7,18^, which changes the value of $\varepsilon$ linearly from 1 to 0.001.

The reward function, *R*, is defined by using the mix-variance, $\Phi$, which is set to be a monotonically decreasing function of $\Phi$ to ensure that the smaller value of $\Phi$ represents a better mixed scalar field:

2 $$\begin{aligned} R(\Phi )=\frac{{\tilde{\Phi }- \Phi }}{\xi },~~~~~~ \xi = {\left\{ \begin{array}{ll} \Phi _{0} - \tilde{\Phi }~~~(\Phi \ge \tilde{\Phi }),\\ \tilde{\Phi }-\Phi _{T}~~~(\Phi < \tilde{\Phi }), \end{array}\right. } \end{aligned}$$

where $\tilde{\Phi }$, $\Phi _{0}$, and $\Phi _{T}$ denote a threshold, an initial value, and a target value of the mix-variance, respectively. By definition, $R=-1$ initially, and $R = +1$ if the mix-variance, $\Phi$, attains the target value. The values of $\tilde{\Phi }$ and $\Phi _{T}$ are set based on the Péclet number: $(\tilde{\Phi },~\Phi _{T})=(1 \times 10^{-2},~4 \times 10^{-3})$ for $\text {Pe} =\infty$ and $(\tilde{\Phi },~\Phi _{T})=(5 \times 10^{-3},~1 \times 10^{-4})$ for $\text {Pe}=100$.

## Results

### RL-based optimization

The optimization results are presented in the absence of diffusion ($\text {Pe}=\infty$). The optimal policy, $\pi ^*: \mathbb {R}^{N_s} \rightarrow {A}$, approximated by the deep Q network, is obtained from the RL-based optimization. Thereafter, the state vector, $s_t \in \mathbb {R}^{N_s}$, determines the optimal action through $\omega _t = \pi ^*(s_t)$. This determines the velocity field during the next interval, $\Delta t_Q$, which advects the scalar field, and the process carries on to the next observation. This flow controller based on the optimal policy, $\pi ^*$, is referred to as a *trained mixer*. Figure 1b shows from the left to right panels that the trained mixer makes the scalar field, *c*(*x*, *t*), evolve in time. Here, the black and white colors correspond to the high and low values of the scalar field, respectively. The trained mixer produces a complex layered structure of the scalar field. The following subsection presents a detailed description of the successive stretching and folding of the interface between the two colors.

The mix-variance, $\Phi _{n}(t)~~(n=1,\ldots , 4000)$, is shown in Fig. 1c. During the initial stage of the training, (i.e., in the first half of the total episodes such as $n=1, 800,$ and 1600), the RL algorithm with the $\epsilon$-greedy method chooses actions randomly. Although this “random mixer” can decrease the mix-variance, such mixing is inefficient, as explained below.

Mathew et al.^2^ reported that the proposed conjugate gradient descent method resulted in $\Phi (t=1) \simeq 6 \times 10^{-3}$; this value of the mix-variance is used for the comparison as a reference. In the first half of the total episodes, the mix-variance at the end of the mixing process, $\Phi _{n}(t=1)$, is larger than the reference value; that is, the insufficient training of the mixer results in inefficient mixing. Conversely, $\Phi _{n}(t=1)$ is reduced in the latter half of the total episodes, $n=2400$, 3200, and 4000. Particularly, $3 \times 10^{-3}< \Phi _n(t=1) < 4 \times 10^{-3}$ for $n=4000$, which are almost identical to (slightly smaller than) the reference value. Interestingly, the mix-variance decreases exponentially fast for $0.3 \le t \le 1$ for the latter episodes such as $n=3200$ and $n=4000$. While here we focus on the *quantitative* comparison using the mix-variance, there are some *qualitative* differences between the method by Mathew et al.^2^ and our RL-based method. In the “Conclusion and discussion” section, we illustrate significant advantages of the RL-based method.

Figure 1d presents the mix-variance at the end of each mixing process, $\Phi _n(t=1)$, which fluctuates due to the $\varepsilon$-greedy methods and the fact that the policy, $Q^{w}$, is not converged. However, the fluctuation decreases as the episode progresses; see also Figs. S1 and S2 in the “Supplementary Information”. The RL algorithm significantly decreases the mix-variance, $\Phi _n(t=1)$; that is, the RL-based optimization effectively enhances the mixing.

### Characteristics of the trained mixer

**Figure 2:**
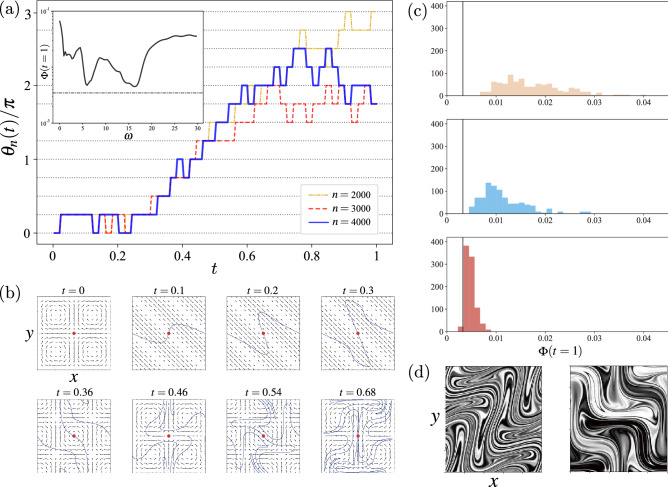
Characteristics of the trained mixer. (**a**) Time series of the flow parameter, $\theta _{n}(t)$, for the *n*-th episode: $n=2000$, 3000, and 4000. The vertical axis is $\theta _{n}(t)/\pi$ and the horizontal dot lines represent $k/4~(k \in \mathbb {Z})$. Inset: the mix-variance, $\Phi (t=1)$, in the case of the flow parameter with the constant angular frequency, $\theta (t)= \omega t$. The horizontal dashed-dotted line indicates the value of the mix-variance by the trained mixer. (**b**) Time evolution of the velocity vector field by the trained mixer. The blue line and the red point represent the material line and one of the fixed points, respectively: $t=0,~0.1,~0.2,~0.3,~0.36,~0.46,~0.54$, and 0.68. (**c**) Probability density functions of the mix-variance, $\Phi (t=1)$, by the completely randomized mixer and the partially randomized mixers I and II from top to bottom panels. (**d**) Scalar fields, $c(x,t=1)$, at the end of the mixing process by the trained mixer (left) and the completely randomized mixer (right).

The flow parameter in episode *n* is denoted by $\theta _{n}(t)$. In the first half of the training, $n<2000$, the flow parameter, $\theta _{n}(t)$, evolves randomly in time due to the $\varepsilon$-greedy methods and the fact that the policy is not converged. However, as the episode progresses, $\theta _{n}(t)$ converges to a single function, $\theta ^*(t)$, except for the final stage of the process, as shown in Fig. 2a. The time series of $\theta _{n}(t)$ consists of square waves, as the velocity field (i.e., $\theta _{n}(t)$) is fixed in each interval, $\Delta t_Q$. The optimal mixing process by the trained mixer corresponding to $\theta ^{*}(t)$ is divided into the following three stages:

1. Initial stage ($0< t \le 0.3$): the flow parameter is a constant; $\theta ^{*}(t) =\pi /4$, indicating the steady flow, $u(x,y)= - c \sin 2 \pi (x+y),~ v(x,y)= c \sin 2 \pi (x+y)~~(c:\text {const.})$. The velocity vector is parallel to the diagonal line; for example, the flow along the line, $x+y=1/4$, traverses the domain, $\mathbb {T}^2$, with the velocity vector $(u,v)=(-c,c)$.
2. Middle stage ($0.3 < t \le 0.7$): the flow parameter changes linearly; $\theta ^{*}(t) = \omega ^{*} t~~(\omega ^{*} \simeq 16)$, indicating the temporally periodic flow with a constant angular frequency.
3. Final stage ($0.7 < t \le 1$): there are no common characteristics of the time evolution of the flow parameter.

The different time evolutions of the flow parameter, $\theta (t)$, in the final stage result in the almost identical value of the mix-variances, $\Phi (t=1)$, at the end of the process. Therefore, the essential process in the mixing is not the final stage but the initial and middle stages. Numerical experiments supporting this point are presented in the next subsection.

Figure 2b presents the time evolution of the velocity fields corresponding to the flow parameter obtained in the final episode, i.e., $\theta _{n}(t)~(n=4000)$. The blue line in each panel indicates the material line advected by the flow, which is initially placed along with the line $y=0.5$. The upper panels in Fig. 2b depict the flow in the initial stage, where the material line is elongated to have the diagonal length of the domain. Subsequently, the flow is temporally periodic in the middle stages, which are shown in the lower panels in Fig. 2b. Each velocity field has eight fixed (stagnation) points, $u_1$ and $u_2$. Half of them are elliptic; that is, the Jacobian matrix has purely imaginary eigenvalues. The other half are saddle points; that is, the Jacobian matrix has real eigenvalues^15,16^. We focus one of them at $(x,y)=(0.5,0.5)$, which is depicted by the red point in each panel of Fig. 2b as a reference. The material line around the fixed point is stretched along the unstable eigendirections when the fixed point is a saddle, whereas it is folded (approximately $\pi /2$ rotation) when the fixed point is elliptic. The local stretching and folding around the eight fixed points occur simultaneously, resulting in efficient mixing. The use of the specific protocol by the trained mixer with the *constant* angular frequency, $\theta (t)=\omega ^{*} t$, is explained in the section of “Conclusion and discussion” section.

Remarkably, the period of the flow in the middle stage, $2\pi /\omega ^*$, which determines the period of the successive switching of the saddle and elliptic types of the fixed points, is optimal in the following sense. Apart from the RL algorithm, we conduct numerical simulations of the scalar field advected by the flow determined by $\theta (t)= \omega t$ with a constant angular frequency, $\omega$, throughout the mixing process, $0\le t \le 1$. The inset of Fig. 2a shows $\Phi (t=1)$ evaluated for $\omega \in [0,30]$. The minimum of $\Phi (t=1)$ in this setting is obtained at $\omega \simeq \omega ^{*}$. This implies that the RL algorithm determines the optimal angular frequency, $\omega ^{*}$, without any prior knowledge, and the trained mixer uses the temporally periodic flow with the optimal period in the middle stage of the process.

### Comparison with randomized mixers

To characterize the flow by the trained mixer in the initial and middle stages, we introduce three different mixing processes, called *randomized mixers*:

- Completely randomized mixer: It uses the random controller that takes one of the three actions, $\omega \in \{ 0, \omega _{+}, \omega _{-}\}$, independently, with the same probabilities for all the stages ($0 \le t \le 1$).
- Partially randomized mixer I: It uses the trained mixer for the initial stage ($0 \le t < 0.3$), and then switches to use the random controller for $0.3 \le t \le 1$.
- Partially randomized mixer II: It uses the trained mixer for the initial and middle stages ($0 \le t < 0.7$), and then switches to use the random controller for $0.7 \le t \le 1$.

Numerical simulations are conducted 200 times independently for each control. Fig. 2c presents the probability density functions (PDFs) of the mix-variance, $\Phi (t=1)$, at the end of the mixing process. The gray solid line indicates the value of the mix-variance by the trained mixer, $\Phi _{n}(t=1)~(n=4000)$ (see Fig. S1 in the “Supplementary Information” for the related PDF of the trained mixer).

The top panel of Fig. 2c depicts the PDF in the case of the completely randomized mixer, where the mix-variances are larger than the reference value of the trained mixer. The left and right panels of Fig. 2d represent the final state of the scalar field, $c(x,t=1)$, produced by the trained mixer and one completely randomized mixer that presents the mix-variance, $\Phi (t=1)$, close to the median value of the PDF. Videos 1 and 2 in the “Supplementary Information” correspond to the scalar fields mixed by the trained mixer and the completely randomized mixer, respectively. Large unmixed blobs remain in the scalar field produced by the completely randomized mixer. That is, the training mixer with the RL algorithm is effective. The second panel of Fig. 2c depicts the PDF in the case of the partially randomized mixer I, which is more effective than the completely randomized mixer. However, a substantial gap exists between the results of the partially randomized mixer I and that of the trained mixer. This indicates that the mixing process during the middle stage is also crucial. Finally, the third panel of Fig. 2c depicts the PDF produced by the partially randomized mixer II. The results are almost identical to those obtained using the trained mixer. Therefore, the effectiveness of the partially randomized mixer II is the same as that of the trained mixer. These observations demonstrate that the mixing process during the initial and middle stages is essential for the mixing efficiency, whereas the mixing process during the final stage is not.

### Transferring the trained mixer

**Figure 3:**
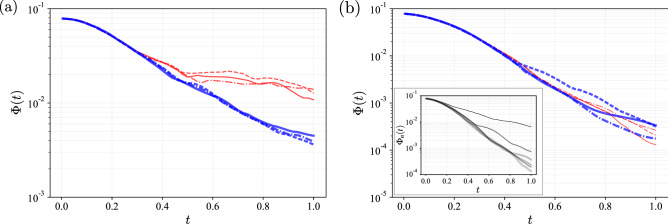
Mix-variance $\Phi (t)$ for $0 \le t \le 1$ at (**a**) $\text {Pe} =\infty$ and at (**b**) $\text {Pe} =100$. The thick blue and thin red lines represent the results of the mixer trained at $\text {Pe}_T =\infty$ and $\text {Pe}_T =100$, respectively. The solid, dashed, and dashed-dotted lines correspond to the results with different random numbers used for the learning. Inset: the mix-variance $\Phi _{n}(t)$ at $\text {Pe} =100$ and $\text {Pe}_{T} = 100$ for the *n*-th episode: $n=1, 600, 1200, 1800, 2400$, and 3000, where lighter (thicker) curves correspond to larger *n*.

This subsection considers the diffusion effect on the RL-optimization of the mixing described by the advection–diffusion equations (Eq. 1) with finite Péclet numbers. The details of the problem settings are identical to those in the previous sections, except for the values of the Péclet numbers. The RL-based optimization is applied to the mixing problem for the case of $\text {Pe}=10^2, 10^3$, and $10^4$, which are as effective as for the case of $\text {Pe}=\infty$, regardless of the Péclet numbers. For instance, at $\text {Pe}=100$, the mix-variance, $\Phi _{n}(t)$, decreases faster for the later episodes, as shown in the inset of Fig. 3b, where $n=1,600,1200,1800,2400$, and 3000 and lighter (thicker) curves correspond to larger *n*. We note that the curves of $\Phi _{n}(t)$ for $n\ge 1200$ are almost the same, implying that the RL algorithm converges to find the optimal policy at $n=1200$. Interestingly, this convergence is faster than the case of $\text {Pe}=\infty$ (Fig. 1c). The number of episodes required for convergence is $n \simeq 3000$ at $\text {Pe}=\infty$; however, $n \simeq 1200$ seems to be sufficient for convergence around $\text {Pe}=100$.

The diffusion effect appears in the flow controls at the later stages. If the mixer successfully generates fine layered structures in an early stage, the flow control becomes less important in the later stages of mixing due to the diffusion effect. In other words, at a low Péclet number, once the RL algorithm finds the optimal mixing control in an early stage of mixing, nothing is to be learned as the diffusion reduces the mix-variance rapidly, irrespective of the control by the mixer. This may result in the faster convergence observed above. The implications of the fast convergence at the low Péclet numbers to the training mixer are given in the “Conclusion and discussion” section.

This diffusion effect implies the *asymmetric transferability* of a trained mixer; that is, a mixer trained at a high Péclet number can be used for mixing at a lower Péclet number, whereas the converse does not hold true. Let $\text {Pe}_T$ be the Péclet number where the mixer is trained, and the asymmetric transferability is then rephrased as follows: the trained mixer can be reused for the same mixing process for the range of $(0,\text {Pe}_T]$. Figure 3a presents the mix-variance, $\Phi (t)$, for $0 \le t \le 1$ at $\text {Pe} =\infty$. The thick blue lines indicate the results for the case of $\text {Pe}_T =\infty$, and the thin red lines indicate the results for the case of $\text {Pe}_T =100$. In Fig. 3a,b, the solid, dashed, and dashed-dotted lines indicate the results with different random numbers for learning. The mixers that trained at $\text {Pe}_T =\infty$ realize the exponentially fast mixing for the entire process when we use it for $\text {Pe}=\infty$. On the other hand, the mixers that trained at $\text {Pe}_T =100$ realize the exponentially fast mixing only for the first half of the process, but fail to mix for the latter half.

Fig. 3b presents the mix-variance, $\Phi (t)$, for $0 \le t \le 1$ at $\text {Pe} =100$. Similar to Fig. 3a, the thick blue lines represent the results for the case of $\text {Pe}_T =\infty$, and the thin red lines represent the results for the case of $\text {Pe}_T =100$. Unlike the case of $\text {Pe}=\infty$, no significant difference exists between the results for the cases of $\text {Pe}_T =100$ and $\text {Pe}_T =\infty$, and both cases realize the exponentially fast mixing. In summary, the mixers of $\text {Pe}_T=\infty$ can be used for the mixing at $\text {Pe}=100$, whereas the converse does not hold true. Therefore, a mixer trained at a higher Péclet number can be used for the mixing process for a broader range of $\text {Pe}$.

## Conclusion and discussion

By illustrating why the RL algorithm is suitable for fluid mixing optimization, we demonstrated, as a proof-of-concept, that the mixer trained by using the RL algorithm is effective for the two-dimensional fluid mixing problem (Fig. 1), which paves the way for the development of RL-based training of mixers. The proposed method was quantitatively evaluated by focusing on the benchmark problem of the mixing optimization studied in the pioneer work^2^. In addition to the comparison of mix-variance values, we note that our RL-based method solves the optimization problem in more restrictive conditions compared to the method proposed by Mathew et al.^2^. For example, in our setting, the number of the states of the velocity field is restricted to eight, $\theta = 0,\pi /4, \ldots , 7\pi /4$. Furthermore, the proposed method is more flexible; that is, it uses only the scalar and velocity field as the input to the neural network. Provided that these fields can be observed, physical implementations are possible in principle, even if the evolution equations of these fields are unknown. For instance, mixing problems of granular or viscoelastic fluids are essential; however, the evolution equation of such a complex material has not necessarily been established, and therefore, the conjugate gradient descent method^2^ cannot be applied to these industrially fundamental problems. On the other hand, the RL based method is equation-free, hence it is applicable if the sensory data of the mixture states are available as the input to the neural network.

The optimized mixing process was divided into three distinct stages. It is particularly interesting to note that, in the middle stage, the optimized flow is temporally periodic with the constant angular frequency. Here, we discuss why the RL algorithm makes the angular frequency constant. The fixed points in both velocity fields, $u_{1}$ and $u_{2}$, are located at the same position and are homogeneously placed in the domain, $\mathbb {T}^{2}$. If the angular frequency is not constant, the switching period between the saddle and elliptic types of the fixed point may differ at each location. This spatial difference makes the scalar field inhomogeneous. The inhomogeneity increases the amplitude of the Fourier coefficient of the small wavenumber, thereby increasing the mix-variance. Consequently, the time variation of the angular frequency results in the larger value of the mix-variance. The RL algorithm employs the constant angular frequency to avoid this undesired effect. The detailed justification of the aforementioned interpretation is one of the future works.

Another related future work is to understand the optimal mixing in more detail. For instance, we claim that the random variation of the flow parameter in the final stage ($t>0.7$) is not essential for optimal mixing, in the sense that the results of the partially randomized mixer II (Fig. 2c) and the trained mixer (Fig. S1 in the “Supplementary Information”) are almost identical. However, there is a small difference between these PDFs, which suggests that the randomization of the actions in the final stage may eliminate some actions, which the RL algorithm regards to be essential, in the optimized mixing process.

For practical application, reduction of learning costs is crucial. Despite the effectiveness of *transfer learning* in reducing learning cost, its application to the problems in fluid mechanics remains limited^19^. In this regard, this study has introduced the physically-reasonable notion of the asymmetric transferability of the trained mixer. The demonstration in this study (Fig. 3) indicates that, in terms of transfer learning, the Péclet number of the *source domain* $\text {Pe}_{T}$ should be as high as possible, if the trained mixer is required to reuse for the broader range. If the mixer is trained at a high Péclet number, it can learn how to mix the scalar field to create the fine striped structures. If the trained mixer is transferred to a lower Péclet number, it makes the fine structures at the beginning of the mixing process. Then, smoothing such structures by diffusion reduces the mix-variance, irrespective of the actions of the trained mixer in the later stage. Therefore, the transfer of the trained mixer from a high Péclet number to a lower one is effective.

Concerning another aspect of learning costs, we have found that the learning of mixing at a *lower* Péclet number converges *faster* (Inset of Fig. 3b). Therefore, if fast learning at a Péclet number is required, the Péclet number of the source domain $\text {Pe}_{T}$ should be as low as possible. Considering together with the discussion in the previous paragraph, the above discussions suggest a trade-off between broad transferability and fast learning; in other words, there is an optimal Péclet number of the source domain that balances these two advantages in each application. Although this study is restricted to the transfer of the trained mixer over the different Péclet numbers, future developments of transfer learning methods of trained mixers may be significant.

Large gaps exist between the mathematical toy problem discussed in this study and the existing mixing problems in the industrial processes. However, the results of this study indicate some directions overcoming these gaps. First, we discuss the implications of this study to turbulent mixing. Turbulence comprises multi-scale counter-rotating pairs of coherent vortices^20^, and strong turbulent mixing stems from the effective mixing around such pairs of vortices at each scale^1^. As observed in the transfer learning method, scalar mixing occurs from larger to smaller scales. Since the time scale of turbulent mixing is shorter for smaller scales, the total mixing efficiency is determined by the mixing at the largest scale. Thus, measuring the velocity and scalar field at the largest scale may be sufficient for the proposed training method. Despite the significant gap between laminar and turbulent mixing, the insights from the present study will be useful for training mixers with turbulent flows.

In addition, in industry, the multiphase and/or thermal flows with chemical reactions may have to be considered, which increases the complexity of the flow dynamics. In such cases, embedding prior knowledge, such as the evolution equations or some physical constraints into the RL-based optimization may be effective, as discussed in Brunton^11^. As another future task for the RL-based optimization in industrial mixing problems, it will be important to study the robustness of the mixing control with the obtained policy with respect to changes in the initial scalar field. Furthermore, whereas the deep Q network is employed as the first step in this study, a more specific and state-of-the-art implementation of the RL algorithm would be necessary for such complex flows. Extending the proposed method to incorporate knowledge on fluid mechanics and suitable RL implementation techniques can further enhance mixing even in industrial processes with laminar and turbulent flows.

## Supplementary Information

Supplementary Information.

Supplementary Video 1.

Supplementary Video 2.

## Data Availability

The datasets used and/or analyzed during the current study are available from the corresponding author on reasonable request.
