# Supplementary material for: Fluid mixing optimization with reinforcement learning
Source: Sci Rep. 2022 Aug 22;12:14268. doi: 10.1038/s41598-022-18037-7 (PMC9395405; doi:10.1038/s41598-022-18037-7)
Supplement: Supplementary file 1 — Supplementary Information. [file 41598_2022_18037_MOESM1_ESM.zip › Supplementary Material_submit/Supplementary Material_final.pdf]

Supplementary Information:
Fluid Mixing Optimization with Reinforcement Learning

Mikito Konishi[1], Masanobu Inubushi[1,2*], and Susumu Goto[1]

[1]Graduate School of Engineering Science, Osaka University, Osaka 560-8531, Japan
[2]Department of Applied Mathematics, Tokyo University of Science, Tokyo 162-8601, Japan

[*]inubushi@rs.tus.ac.jp

1 Details of the network structure and RL algorithm

We employ the deep Q network and the training algorithm which are the same as those used in Mnih et al. (2015) with slightly different sizes of filters and parameters. The network used in this study consists of the three convolutional layers and two fully connected layers with the ReLU activation function. In more detail, the size of the input data to the network is $83 \times 83 \times 7$ as written in the section *Optimization of Mixing with RL* in the main text. The first hidden layer convolves 32 filters of $8 \times 8 \times 7$ with stride 4 with the input data. The second hidden layer convolves 64 filters of $4 \times 4 \times 32$ with stride 2. The third hidden layer convolves 64 filters of $3 \times 3 \times 64$ with stride 1. Then, we have a 7744 ($= 11 \times 11 \times 64$) dimensional vector, calculate the matrix-vector product with the weight matrix with the size of 256×7744, and apply the ReLU function. The output layer operates the the weight matrix with the size of 3×256, and finally, we have a three-dimensional vector, which represents the value corresponding to the selected action. We employ the stochastic decent algorithm with the minibatch size 32 and learning rate 10^{-4} used in the Adam optimizer, and the experience replay with the memory size 10^4.

2 Convergence of the learning process

We use the value of the mix-variance of the final episode, $\Phi_n(t = 1)$ with $n = 4000$, as a reference in Fig. 2 (c) (the gray solid line) in the main text. Since the mix-variance fluctuates during the final stage of the episodes ($3600 < n \leq 4000$), we here evaluate the convergence of the learning process. To this end, we compute the probability density function (PDF) by using the data of the mix-variance during the final 400 episodes, i.e., $\Phi_n(t = 1)$ $(3600 < n \leq 4000)$, where the policy almost converges. The PDF shown in Fig. S1 suggests that the learning process converges enough, and the final mix-variance,

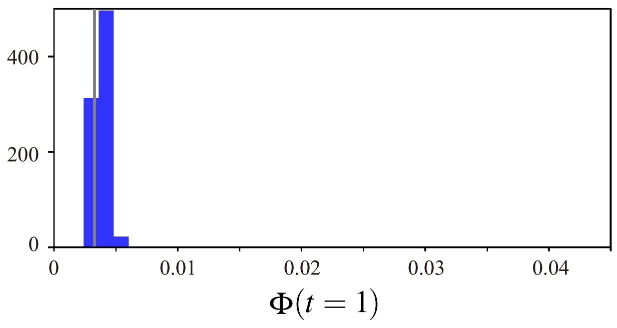

Figure S1 Probability density functions of the mix-variance $\Phi_n(t = 1)$ for $3600 \leq n \leq 4000$. The gray solid line indicates the final value of the mix-variance, $\Phi_n(t = 1)$ $(n = 4000)$.

$\Phi_n(t=1)$ $(n=4000)$, does give a typical value during the final stage of the episodes.

3 Independence of the optimization process on the realization of the random numbers

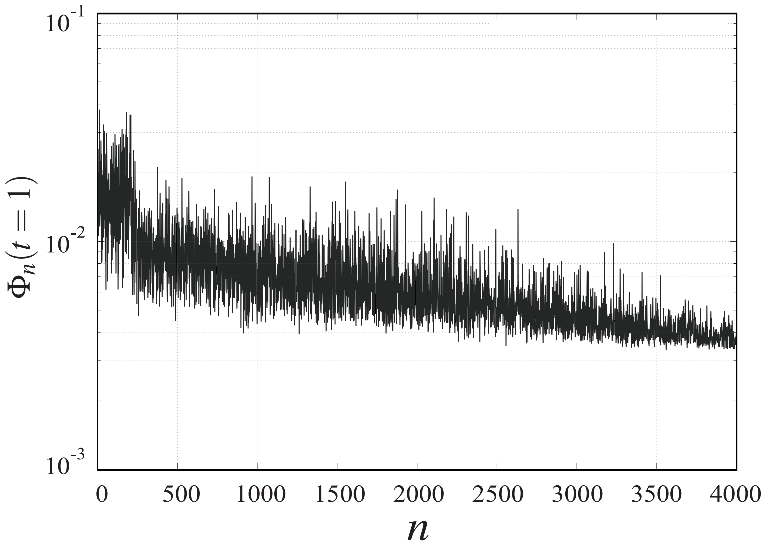

Figure S2 Mix-variance, $\Phi_n(t=1)$, at the end of each episode.

We show another realization of the training process $\Phi_n(t=1)$ in Fig. S2, by using a seed value of the random number generator that is different from the one used in the main text. The selection of the seed determines the initial weight of the deep Q network and random numbers used in the ϵ-greedy methods. The training process shown in Fig. S2 is similar to that shown in Fig. 1 (d) in the main text, indicating that our results are independent of the realization of the random numbers used in the algorithm.
