# Supplementary figures and images for: Fluid mixing optimization with reinforcement learning
Source: Sci Rep. 2022 Aug 22;12:14268. doi: 10.1038/s41598-022-18037-7 (PMC9395405; doi:10.1038/s41598-022-18037-7)

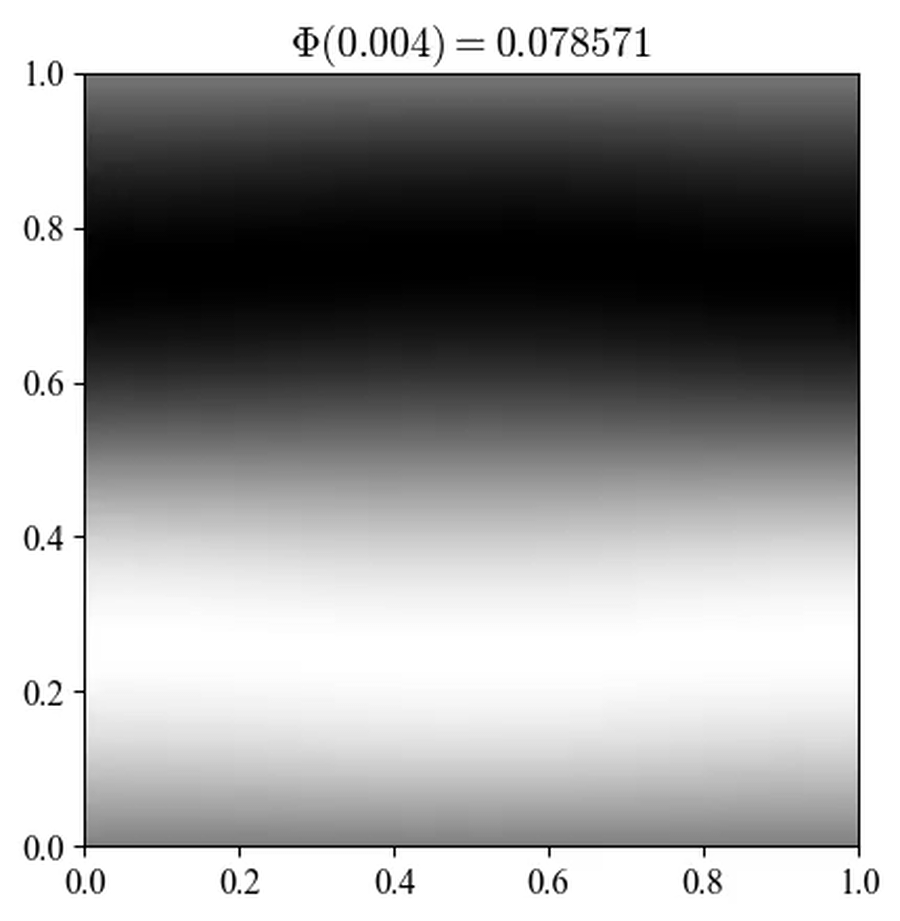

Supplement: Supplementary file 2 — Supplementary Video 1. [file 41598_2022_18037_MOESM2_ESM.gif]

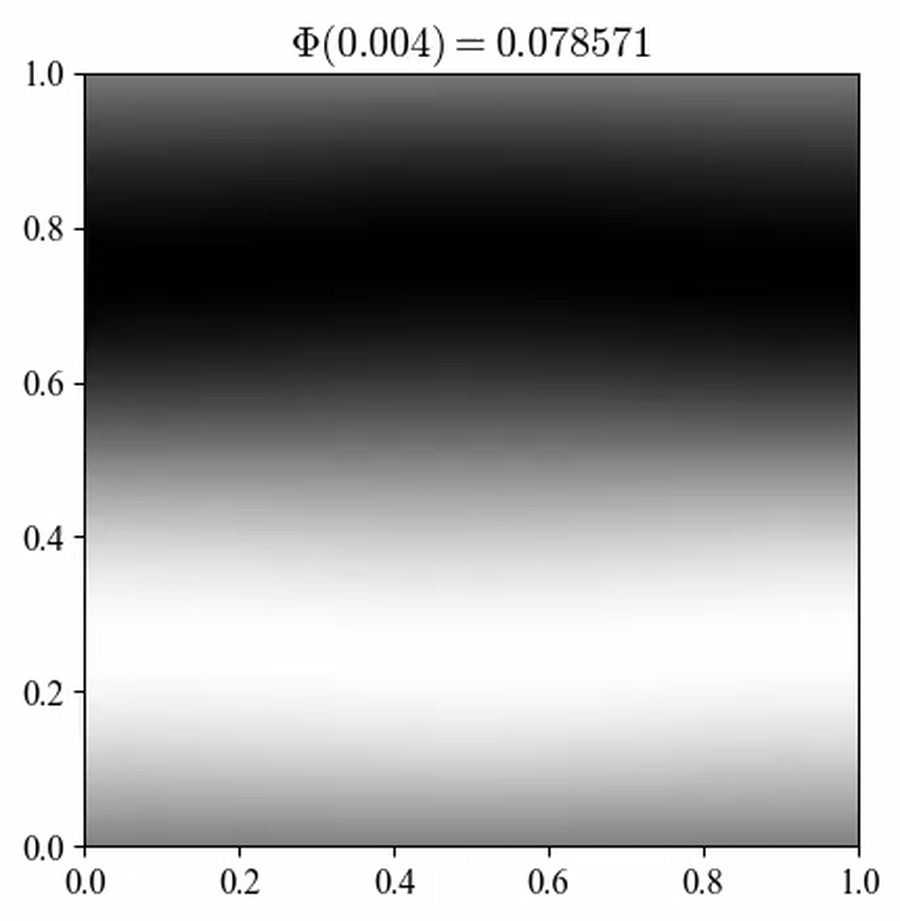

Supplement: Supplementary file 3 — Supplementary Video 2. [file 41598_2022_18037_MOESM3_ESM.gif]
